# The European Journal of Ageing in the European Year for Active Ageing

**DOI:** 10.1007/s10433-012-0218-8

**Published:** 2012-02-14

**Authors:** D. J. H. Deeg, H.-W. Wahl

**Affiliations:** 1 VU University Medical Centre/LASA, Van der Boechorststraat 7, 1081 BT, Amsterdam, The Netherlands; 2 Department of Psychological Ageing Research, Institute of Psychology, University of Heidelberg, Bergheimer Strasse 20, 69115 Heidelberg, Germany

The European Commission has marked the year 2012 as the European Year for Active Ageing and Solidarity between Generations. This action indicates an increased awareness of the urgency to make progress in finding solutions to what some perceive as the “problems” of ageing societies. However, ageing societies also present opportunities for Europe. Based on the premise that Europeans are living longer and staying healthier than ever before, the European Commission states that the time has come to realise these opportunities. Hence, the main aims of this year are stated as: maintaining the vitality of older people, enhancing their involvement in society and removing barriers between generations. The emphasis clearly is on employability and workability, given the dramatic demographic shifts in the workforce all over Europe, but also on living independently, health care, social services, adult learning, volunteering, housing, IT services and transport. Under the flag of Europe, a series of initiatives are launched, ranging from conferences and events, information campaigns to exchange of information and best practice. Many national, regional and local authorities as well as social partners and businesses in Europe launch initiatives in parallel. These initiatives should raise awareness, stimulate debate and have a real impact on fostering a sustainable active ageing culture. All initiatives can be found on the official website http://europa.eu/ey2012.

An important initiative is the pilot European Innovation Partnership on Active and Healthy Ageing. This partnership has a triple aim:

1. Enabling EU citizens to lead healthy, active and independent lives whilst ageing.
2. Improving the sustainability and efficiency of social and health care systems.
3. Boosting and improving the competitiveness of the markets for innovative products and services, responding to the ageing challenge at both EU and global level, thus creating new opportunities for businesses.

This triple aim should be realised by 2020 in the three policy areas of prevention and health promotion, integrated care and independent living of older people.

To achieve such aims, it is important to have research-based evidence available and also to know where such evidence is still lacking. In October 2011, a Road Map for Future Ageing Research in Europe has been presented to the European Parliament as the outcome of the 2-year coordinated action FUTURAGE (http://futurage.group.shef.ac.uk/). The Roadmap claims to be the most comprehensive effort ever undertaken to further European ageing research, striving to set research priorities for the next 10–15 years in all major areas from biogerontology and the health sciences to the behavioural and social sciences.

The trade of the European Journal of Ageing is the dissemination of research findings. Now in its 9th volume, the Journal plays an increasing role as an outlet of research reports from Europe and other parts of the world including North America and Asia. Thus, it potentially helps to inform debates and best practices. This dissemination is increasingly effective as the Journal gains visibility. We as editors are happy to note that the Journal’s visibility is certainly growing. For example, last year we saw an almost doubling of our impact factor (from 0.61 to 1.11), and we are confident that it will continue to rise. Amongst the 28 Gerontology journals included in the Social Science Citation Index, the rank of our Journal rose from 23rd in 2009 to 15th in 2010. The number of full text downloads shows a steady growth, as does the number of subscribers to Table of Contents alerts. In parallel with these developments, the number of submissions is growing, and the publisher has allowed the Journal 30% more pages.

We realise that the viability of our Journal rests on the contribution of both authors and reviewers. A special role is for the Editorial Board, whose members help to determine the policy of the Journal and serve with high-quality reviews of submitted articles. In order to keep the Editorial Board effective, we felt that it should include a fair share of early-career researchers. Thus, during the past year, we renewed one-third of its members. In this issue, we welcome all new members, and send our heart-felt thanks to the members who have left the Board after having contributed so much to our Journal. The new Editorial Board can be found on the last page of this issue.

The current issue very appropriately sets the stage for the European Year of Active Ageing and Solidarity between Generations with a special section on ‘Comparative Contexts of Care: Findings from the Survey of Health, Ageing and Retirement in Europe’—for an introduction, see the contribution from guest editor Howard Litwin (2012). Whilst the data for the Survey of Health, Ageing and Retirement in Europe (SHARE) are collected mainly in western European countries, still a lot needs to be learned about Eastern Europe. Therefore, the article by Nikolai Botev (2012), titled ‘Population Ageing in Central and Eastern Europe and its Demographic and Social Context’ in the section Critical Positions in Ageing Research is a very timely contribution. From demographic data, the author shows developments in individual eastern European countries that are often very different from those in western European countries. The article makes a strong case against the tendency to extrapolate developments from western to eastern European countries, as well as the tendency to generalise across all eastern European countries. In particular, the article provides ample evidence that contradicts the premise for the European Year of Active Ageing: ‘Europeans are living longer and staying healthier than ever before’. Certainly, such a wake-up call leads to new research questions, several of which are proposed in the article.

As a contrast to all the statistical evidence from survey and demographic data reported in the articles described so far, this issue includes one report from a qualitative study of people’s responses to being notified that their pattern of nutrition holds health risks (Reimer et al. 2012). The European Journal of Ageing receives an increasing number of submissions based on qualitative studies. We feel that this is a good development, because it enables the Journal to show the breadth and depth of the full range of ageing research from social, behavioural and health perspectives. Moreover, both types of research, quantitative and qualitative, are likely to contribute their share to an informed debate and policy measures that have a real impact—the exact aims of the European Year for Active Ageing and Solidarity between Generations.
